# Supplementary figures and images for: A validation of Emotiv EPOC Flex saline for EEG and ERP research
Source: PeerJ. 2020 Aug 11;8:e9713. doi: 10.7717/peerj.9713 (PMC7427545; doi:10.7717/peerj.9713)

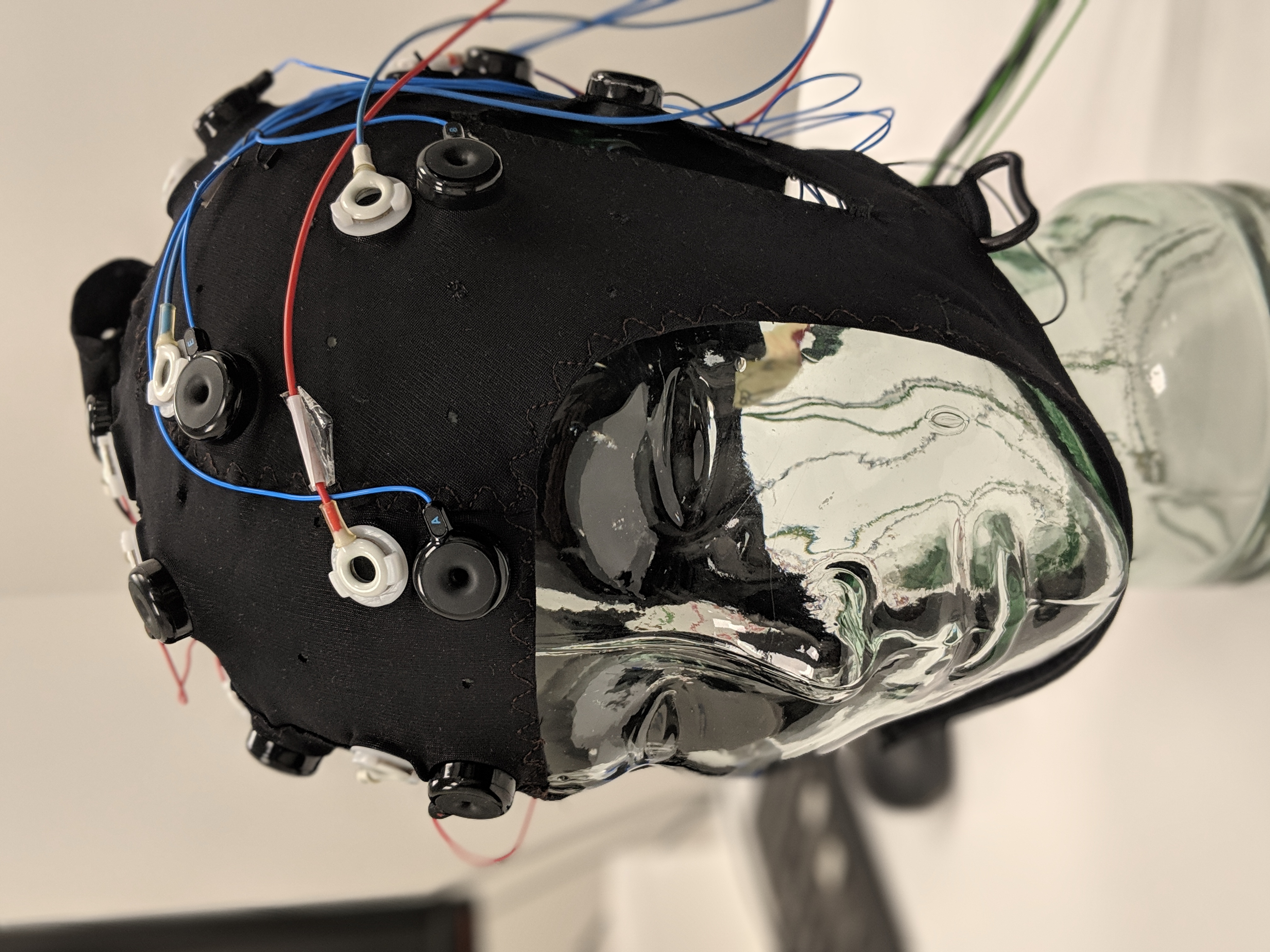

Supplement: Supplemental Information 1 [file peerj-08-9713-s001.jpg]
